# Evaluating the Need for Durotomy and Duraplasty in Adults Undergoing Suboccipital Craniectomy for Chiari Decompression: A Case Series Analysis of Radiographic and Clinical Outcomes

**DOI:** 10.7759/cureus.60694

**Published:** 2024-05-20

**Authors:** Rohan Jha, Joshua I Chalif, Yi Lu

**Affiliations:** 1 Neurosurgery, Harvard Medical School, Boston, USA; 2 Neurosurgery, Brigham and Women's Hospital, Boston, USA

**Keywords:** ccos, csf, duraplasty, sub-occipital craniotomy, chiari 1 malformation

## Abstract

Background

Suboccipital craniectomy (SOC) in conjunction with dura opening and duraplasty for posterior fossa decompression is an effective treatment for symptomatic Chiari 1 malformations (CM1), primarily carried out in the pediatric population. However, dural opening and reconstruction are associated with an increased risk of complications, and their necessity in the adult population has not yet been robustly demonstrated. Given differences in clinical presentation and disease severity between the pediatric and adult patients, we aimed to identify if SOC alone with intraoperative ultrasound confirmation of adequate restoration of pulsatile motion of cerebellar tonsil is sufficient to treat symptomatic CM1 while mitigating surgical risks.

Methods

We identified a retrospective, institutional cohort of adult patients who underwent SOC for Chiari decompression between 2014 and 2023. Demographic, clinical, and radiographic features were extracted for each patient. Clinical outcomes were assessed using the Chicago Chiari Outcome Scale (CCOS) and Motor-Sensory-Sphincter signs score (Clinical Sign Score (CSS)). Radiographic outcomes assessed cerebellar ectopia and associated syrinx characteristics.

Results

A total of 15 patients were identified, with an average follow-up period of three years. Eight patients underwent SOC with duraplasty, whereas seven patients underwent SOC only without duraplasty. Both groups of patients were of similar age at surgery and had similar nature and duration of symptoms prior to surgery. On pre-operative radiographic evaluation, both groups of patients had similar lengths of cerebellar ectopia (9.9±11.0 mm to 11.1±5.7 mm, p=0.591), and associated syrinxes (75% vs. 42.9%, p=0.205). Intraoperatively, both groups had similar estimated blood losses, though the length of surgery was significantly shorter when durotomy was spared (202±58.3 minutes to 116.3±47.8 minutes, p=0.011). The length of ICU stay was also significantly longer in the durotomy group (1.1±0.6 days to 0.0 days, p<0.001). Neither group reported any post-operative complications. On follow-up, both groups demonstrated similar reductions in cerebellar ectopia and syrinx characteristics. Clinically, the CCOS and CSS scores were similar between the two cohorts at follow-up, with no repeat surgery required in either group.

Conclusion

Our cohort suggests that for adult CM1 patients, SOC decompression alone without dural reconstruction might lead to comparable clinical and radiographic outcomes to SOC decompression with durotomy/duraplasty, especially if intraoperative ultrasound confirms good cerebrospinal fluid (CSF) flow after SOC. Notably, sparing durotomy and duraplasty is also associated with decreased operative time and decreased ICU stay.

## Introduction

Chiari I malformation (CM1) is a congenital neurologic hindbrain disorder, where the cerebellar tonsils extend below the level of the foramen magnum. This descent into the spinal canal is often accompanied by the alteration of the normal cerebrospinal fluid (CSF) flow in the craniocervical junction (which can progress to complete CSF block) and compression of the brainstem, spinal cord, vessels, and cranial nerves [1]. Radiologically, the definition is often cited as the elongation and herniation of the cerebellar tonsils of 5 mm or more below the foramen magnum, though the designation is somewhat arbitrary [2]. The incidence has been reported to be 0.5%-3.5% in the general population and 0.56%-0.77% in MRI studies [3].

Classically, CM1 symptoms are described as posterior head and neck pain worsened by rapid increases in abdominal pressure, i.e., occipital tussive headaches [4]. The most common presenting symptoms include headaches and paresthesias, though other often reported symptoms include nausea, dysphagia, cerebellar symptoms, and dysphonia [5]. Nevertheless, many patients remain asymptomatic, as many cases are found incidentally on MRI [6]. In conjunction with the variable range of clinical presentations, there is no consensus on the exact indications for surgery, with little clear evidence to guide management [5]. The severity of symptoms, impact on quality of life, and potential for surgical complications are all factors to be balanced. Asymptomatic patients with only radiologic findings are generally not operated on, though practices vary between clinicians [7]. Commonly used indications for surgery include cough-associated headaches that have an enduring impact on quality of life, the presence of a large or enlarging syrinx (clinician dependent), and/or abnormal neurological findings [8]. 

Symptoms ascribed to CM1 (and CM1.5, which represents a progression of CM1 with additional descent of the brainstem through the foramen magnum) are thought to be due to compression of neural structures and associated CSF disturbances. If surgery is to be performed to alleviate these symptoms, the preferred surgical approach is suboccipital craniectomy (SOC) to expand the posterior cranial fossa [9], in hopes of decompressing and improving the passage of CSF flow in the craniocervical junction [10]. Two commonly used methods include posterior fossa decompression alone, and posterior fossa decompression with duraplasty [11]; both have been widely proven as efficient treatment strategies [12]. Decompression with duraplasty is the treatment of choice in most reported pediatric and adult case series [3]. Dural opening and duraplasty are associated with increased CSF-related complications, including CSF leaks, meningoceles, and meningitis, as well as prolonged ICU and hospital stay [13,14]. However, despite these increased risks, duraplasty is generally thought to be necessary to achieve proper decompression and favorable clinical outcomes [4]. Insufficient decompression leads to symptom persistence and requires revision surgery with dural reconstruction, a complication clinicians prefer to avoid. 

Intraoperative ultrasound can be used in conjunction with decompression without duraplasty to check for the adequacy of decompression. These high-frequency probes are able to clearly visualize the anatomy and CSF flow behind the cerebellar tonsils after bony decompression. If sufficient decompression is achieved, the intraoperative ultrasound should be able to identify the re-expansion of the cisterna magna and pulsations of the cerebellar tonsils [15], indicating the restoration of the CSF flow. 

Although it is widely accepted in pediatric populations to perform a duraplasty [3], there are differences in the CM1 presentation between pediatric and adult populations [4]. Adults often present solely with headaches, and often present with delayed symptoms associated with less-compromised CSF circulation [4]. The adult dura also has differences in distensibility [4]. These differences justify a more nuanced understanding of CM1 as the disease process, and therefore the treatment likely should be different between adult and pediatric populations. In particular, it is unclear if duraplasty is still needed in adult CM1 patients or whether SOC alone is adequate, especially if the intraoperative ultrasound could confirm the restoration of CSF flow and the decompression of the cerebellar tonsil at the craniocervical junction.

Multiple meta-analyses have been conducted, though mostly focused on the pediatric population, to identify the surgical and clinical outcomes associated with duraplasty in CM1 surgical decompression. No consistent outcomes have been identified with respect to symptom improvement [4,11,16,17]. Given these conflicting reports, that they have been focused on the pediatric population, and that the disease presentation and severity differ in the adult and pediatric populations, we sought to examine the clinical impact of dura sparing in SOC decompression for adult CM1 patients, especially after ultrasound confirmation of good CSF flow.

## Materials and methods

Patient cohort

We identified adult patients with symptomatic CM1, who underwent SOC at Brigham and Women’s Hospital (BWH), Boston, United States, between 2014 and 2023 by the senior author (YL). Criteria for surgery included radiographic evidence of CM1 (> 5 mm caudal descent of cerebellar tonsils through the foramen magnum) in conjunction with persistent symptomology, refractory to conservative treatment for at least two months, where surgery was thought to reasonably provide decompression and symptom improvement. To be included in the study, patients had to have a brain MRI prior to and after surgery. Prior decompression and associated syrinxes were not in the exclusion criteria. Patients were excluded if they had asymptomatic, isolated CM1, or associated bony abnormalities of the craniovertebral junction (CVJ), including basilar invagination, Klippel-Feil abnormality, prior surgical fusion, platybasia, and atlas assimilation.

Patient clinical features, including demographic features, were extracted from the electronic medical health record. Pre-operative considerations, including presenting signs and symptoms, neurological deficits, and their associated duration were identified. Intra-operative and post-operative data, including surgical and medical complications, were collected.

Surgical approach

**Figure 1:**
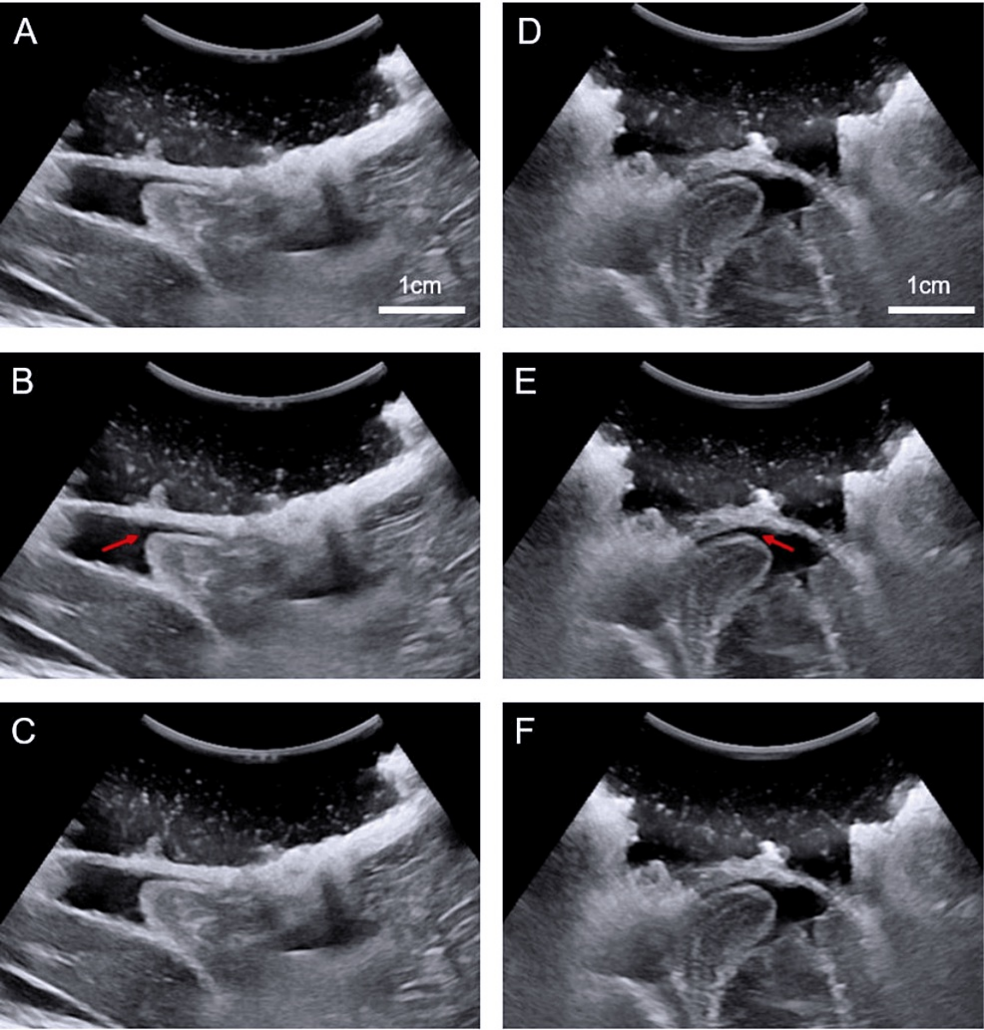
Ultrasound confirmation of cerebellar tonsil decompression with restoration of tonsillar pulsatile motion and cerebrospinal fluid (CSF) flow. A-C: After suboccipital decompression, there is restoration of tonsillar pulsatile motion and CSF flow, with each image representing three different points. The red arrow points to increased CSF flow and distance between the dura and cerebellar tonsil due to tonsillar motion. Sagittal view. D-F: Same figures as in A-C for a different patient.

Suboccipital craniectomy and C1 laminectomy were carried out in a standard fashion in all patients. Intraoperative ultrasound was used to confirm adequate CSF pulsatile flow and tonsillar movement (Figure 1). Determination of adequate flow was carried out by senior author YL. If CSF flow was confirmed, no durotomy and duraplasty were carried out. However, if insufficient flow was encountered, a Y-shaped dura opening was created, and CSF was released from the cisterna magna with the opening of the arachnoid membrane. Expansile duraplasty was subsequently carried out, with AlloDerm (Biohorizons Implant Systems, Inc., Birmingham, Alabama, United States) used to close the dura in a water-tight fashion.

Radiographic evaluation

**Figure 2:**
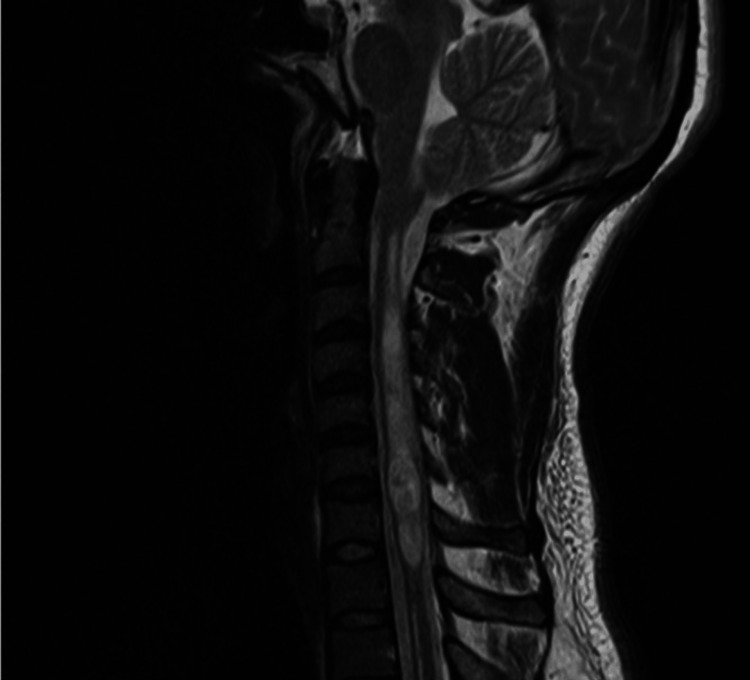
Representative preoperative MRI of a patient demonstrating cerebellar ectopia as well as associated spinal cord syrinx.

Data from the pre-operative MRI closest to the data of surgery was extracted, along with data from MRIs at the first follow-up (generally at about one month) (Figure 2). The length of cerebellar ectopia, as measured by the tonsillar descent orthogonal to the line drawn between the basion and opisthion (McRae’s line) was calculated [18]. Associated syrinx characteristics, if present, were evaluated including syrinx maximum diameter, maximum length, and maximal ratio to spinal cord diameter.

Pain and narcotic pain medication use evaluation

Individual pain-scale ratings were collected from post-operation day (POD)1 and POD2. Due to the variable number of ratings collected each day, the median for each day was collected. Pain scales were also collected at the one-month post-operative visit. Total inpatient morphine equivalents administered following transfer to the post-operative recovery unit were also recorded.

Clinical follow-up evaluation

Symptomology at clinic follow-up was assessed with two scales, including the Chicago Chiari Outcome Scale (CCOS) [19], and Clinical Sign Score (CSS). In the CCOS, the patient’s symptom progression was evaluated with respect to categories of pain, non-pain, functionality, and complications. In the CSS score, patients were evaluated on the basis of motor, sensory, and/or the presence of sphincter signs.

Statistics

Unless otherwise indicated, all data is presented as mean ± standard deviation (SD). Unpaired two-tailed Student’s t-tests were employed to compare sample means between cohorts for both clinical and radiographic measures. All statistical analyses were completed in R 4.3.1 (Posit, PBC, Boston, Massachusetts, United States), and p ≤ 0.05 was used to define statistical significance.

## Results

Duraplasty and dura intact cohorts were similar preoperatively: clinically and radiographically

A total of 15 patients were identified, with eight in the duraplasty cohort and seven in the duraplasty sparing (dura intact) cohort, who were followed for an average of three years (Table 1). Pre-operative clinical presentation data was not statistically different between the two groups, except for the age of surgery. The majority of cases were female (100% duraplasty, 86% intact, p = 0.268), with patients undergoing surgery at an average of 51.6 ± 11.6 years in the duraplasty cohort and 31.1 ± 12.1 years in the dura intact cohort (p = 0.034). Duraplasty and dura intact patients were symptomatic for an average of 4.8 ± 14.2 months and 17.6 ± 16.4 months (p = 0.105), respectively. Tussive headaches were the most frequently reported symptoms (75% and 71.4%), with varying numbers of other symptoms including ocular symptoms, neck pain, sensory abnormalities, motor deficits, extremity pain, and gait/balance issues. One patient had undergone prior decompression in the duraplasty cohort, whereas none had in the dura intact cohort. Concurrent scoliosis was not identified in either population.

**Table 1:** Pre-operative radiographic and clinical data of patients in duraplasty and dura intact cohorts. No statistically significant differences were found in each of the evaluated features between the two cohorts. Unpaired t-tests were used to assess for significance for continuous variables, whereas the chi-squared test for significance was used for discrete variables between the two cohorts. All statistical significance was set at <0.05.

Clinical data	Duraplasty (n = 8)	Dura intact (n = 7)	p-value
Sex (F, n, %)	8 (100%)	6 (86%)	0.268
Age at surgery (years, mean ± std)	51.6 ± 11.6	31.1 ± 12.1	0.034
Symptom duration (months, mean ± std)	4.8 ± 14.2	17.6 ± 16.4	0.105
Tussive headaches? (n, %)	6 (75%)	5 (71.4%)	0.876
Ocular pain? (n, %)	2 (25%)	1 (14.3%)	0.605
Neck pain? (n, %)	4 (50%)	2 (28.6%)	0.398
Sensory abnormalities? (n, %)	5 (62.5%)	1 (14.3%)	0.057
Motor deficits? (n, %)	2 (25%)	1 (14.3%)	0.605
Extremity pain? (n, %)	4 (50%)	1 (14.3%)	0.143
Gait/balance issues? (n, %)	3 (37.5%)	0 (0%)	0.070
History of prior decompression (y, %)	1 (12.5%)	0 (0%)	0.333
Radiographic data	Duraplasty (n = 8)	Dura intact (n = 7)	p-value
Length of cerebellar ectopia (mm, mean ± std)	9.9 ± 11.0	11.1 ± 5.7	0.591
Presence of syrinx (y, %)	6 (75%)	3 (42.9%)	0.205
Syrinx max, diameter (mm, mean ± std)	5.9 ± 3.4	6.4 ± 2.0	0.768
Syrinx max, length (mm, mean ± std)	84.1 ± 75.5	137.7 ± 26.3	0.273
Syrinx to spinal cord ratio, diameter (ratio, mean ± std)	0.59 ± 0.30	0.68 ± 0.08	0.580
Presence of scoliosis (y, %)	0 (0%)	0 (0%)	-

Radiographically, the length of cerebellar ectopia and associated syrinx characteristics, if present, were not statistically different. The average length of cerebellar ectopia was 9.9 ± 11.0 mm in the duraplasty cohort and 11.1 ± 5.7 in the dura intact cohort. Syrinxes were found in 75% of duraplasty patients and 43% of dura intact patients (p = 0.205). Of these syrinxes, the maximum diameter was 5.9 ± 3.4 mm in the duraplasty cohort and 6.4 ± 2.0 mm in the dura intact cohort (p = 0.768). The maximum length was 84.1 ± 75.5 mm in the duraplasty cohort and 137.7 ± 26.3 mm in the dura intact cohort (p = 0.273). Finally, the syrinx to spinal cord diameter ratio was 0.59 ± 0.30 in the duraplasty cohort and 0.68 ± 0.08 in the dura intact cohort (p = 0.580).

Duraplasty is associated with longer surgeries and increased ICU length of stay

The length of surgery was significantly shorter in the dura intact cohort (116.3 ± 47.8 minutes versus 202 ± 58.3, p = 0.011) (Table 2). The estimated blood loss was similar (101.4 ± 176.4 mL versus 156.3 ± 114.8, p = 0.461), as was the average hospital length of stay (2.3 ± 1.3 days versus 3.1 ± 1.1, p = 0.195). Notably, patients with duraplasty had longer ICU stays, with an average stay of 1.1 ± 0.6 days, compared to zero days in the dura intact cohort (p < 0.001). Patients in neither group reported any major medical or surgical complications, including CSF leaks, meningitis, wound infections, sepsis, infarction, pulmonary complications, urinary/renal complications, and thrombosis.

**Table 2:** Operative features of patients in duraplasty and dura intact cohorts. Evaluated post-operative complications included CSF leak, meningitis, wound infection, sepsis, infarction, pulmonary complications, urinary/renal complications, and thrombosis. Unpaired t-tests were used to assess significance for continuous variables, whereas the chi-squared test for significance was used for discrete variables between the two cohorts. All statistical significance was set at <0.05.

Operative feature	Duraplasty (n = 8)	Dura intact (n = 7)	p-value
Length of surgery (minutes, mean ± std)	202 ± 58.3	116.3 ± 47.8	0.011
Estimated blood loss (mL, mean ± std)	156.3 ± 114.8	101.4 ± 176.4	0.461
Hospital length of stay (days, mean ± std)	3.1 ± 1.1	2.3 ± 1.3	0.195
ICU length of stay (days, mean ± std)	0.0 ± 0.0	1.1 ± 0.6	<0.0001
Post-operative complications (y, %)	0 (%)	0 (%)	-

Radiographic outcomes were similar in duraplasty and dura intact cohorts

At one-month post-operative clinical follow-up, the radiographic evaluation suggested similar levels of decompression. Though surgical disruption of the posterior rim of the foramen magnum was precluded using the standard McRae’s line post-operatively, the length of cerebellar ectopia was proxied with the tonsillar-C3 distance, as has been previously published [20]. The duraplasty cohort saw a proxied cerebellar ectopia of 3.5 ± 3.6 mm, whereas dura intact saw 4.5 ± 0.8 mm, which was not statistically significant between the two populations (p = 0.564) (Table 3). When compared to their pre-operative cerebellar ectopia, duraplasty and dura intact patients saw a difference of -5.7 ± 2.6 mm and -4.9 ± 5.2 mm, respectively (p = 0.768). Only one syrinx resolved completely, which was in the duraplasty cohort, with partial resolution of all other syrinxes. Of the persistent syrinxes, the change in maximum diameter was -5.7 ± 2.6 mm in the duraplasty cohort and -4.9 ± 5.2 mm in the dura intact cohort (p = 0.656). The change in maximum length was -31 ± 24 mm in the duraplasty cohort and -18 ± 14 mm in the dura intact cohort (p = 0.131). Finally, the change in syrinx to spinal cord diameter ratio was -0.3 ± 0.4 in the duraplasty cohort and -0.2 ± 0.1 in the dura intact cohort (p = 0.645).

**Table 3:** Radiographic follow-up data. No statistically significant differences were found between the groups for each of the evaluated features, except for the syrinx to spinal cord ratio. All values are reported as mm ± std unless otherwise indicated. Unpaired t-tests were used to assess significance for continuous variables, whereas the chi-squared test for significance was used for discrete variables between the two cohorts. All statistical significance was set at <0.05.

Radiographic follow-up	Duraplasty (n = 8)	Dura Intact (n = 7)	p-value
Time to first follow-up (days, mean ± std)	17.7 ± 8.1	27.1 ± 10.1	0.349
Length of cerebellar ectopia (mm, mean ± std)	3.5 ± 3.6	4.5 ± 0.8	0.564
Δ cerebellar ectopia (mm, mean ± std)	-5.7 ± 2.6	-4.9 ± 5.2	0.768
Syrinx resolved? (y, %)	1 (17%)	0 (0%)	0.056
Syrinx max, diameter (mm, mean ± std)	2.2 ± 0.4	5.2 ± 1.6	0.119
Δ syrinx max, diameter (mm, mean ± std)	-5.7 ± 2.6	-4.9 ± 5.2	0.656
Syrinx max, length (mm, mean ± std)	55.5 ± 20.5	111 ± 60.8	0.346
Δ syrinx max, length (mm, mean ± std)	-31 ± 24	-18 ± 14	0.131
Syrinx to spinal cord ratio, diameter (ratio, mean ± std)	0.35 ± 0.1	0.56 ± 0.2	0.005
Δ syrinx to spinal cord ratio, diameter (ratio, mean ± std)	-0.3 ± 0.4	-0.2 ± 0.1	0.645

Pain and narcotic pain medication usage was higher, but not significant, in the duraplasty cohort

On POD1, the duraplasty cohort reported higher pain values than the dura intact cohort, but the difference was not significant (5.9 ± 2.1 versus 5.4 ± 1.6, p = 0.678). On POD2, the cohorts reported similar trends (4.4 ± 1.5 versus 4.3 ± 2.9, p = 0.977). At one month follow-up, similar trends were observed (4.8 ± 3.7 versus 4.3 ± 2.9, p = 0.380). With respect to narcotic pain medication usage, the duraplasty cohort had higher total inpatient morphine equivalents administered, though the difference was not significant (135.4 ± 95.9 versus 63 ± 58.4, p = 0.114).

Clinical outcomes were also similar in duraplasty and dura intact cohorts.

At the most recent follow-up, with respect to the CCOS, most patients reported predominantly resolved or improved symptoms across all four domains, in both cohorts (Table 4). However, given the number of dimensions in the CCOS score and our limited cohort size, robust statistical analysis was unable to be completed. Nevertheless, with respect to pain and non-pain dimensions, four patients in each cohort were categorized as resolved. With respect to functionality, five patients in duraplasty and six patients in dura intact were categorized as fully functional. Finally, all patients were reported as uncomplicated with respect to complications. For the CSS score, patients saw similar scores (0.4 ± 0.5 versus 0.3 ± 0.5, p = 0.611). Patients in neither cohort required repeat surgery for decompression, nor did they report long-term surgical or medical complications.

**Table 4:** Clinical follow-up data. Limited number of cases across four categories precluded statistical analyses for CCOS scores.

Chicago Chiari Outcomes Scale (CCOS)		Duraplasty (n = 7)	Dura intact (n = 7)
Pain	Resolved	4 (57.1%)	4 (57.1%)
	Improved	2 (28.6%)	3 (43.9%)
	Unchanged	1 (14.3%)	0 (0%)
	Worse	0 (0%)	0 (0%)
Non-pain	Resolved	4 (57.1%)	4 (57.1%)
	Improved	2 (28.6%)	3 (42.9%)
	Unchanged	1 (14.3%)	0 (0%)
	Worse	0 (0%)	0 (0%)
Functionality	Fully functional	5 (71.4%)	6 (85.7%)
	Mild impairment	1 (14.3%)	0 (0%)
	Moderate impairment	1 (14.3%)	1 (14.3%)
	Full impairment	0 (0%)	0 (0%)
Complications	Uncomplicated	7 (100%)	7 (100%)
	Transient	0 (0%)	0 (0%)
	Persistent	0 (0%)	0 (0%)
	Existent	0 (0%)	0 (0%)
Clinical Sign Score (CSS)		0.4 ± 0.5	0.3 ± 0.5
Repeat surgery required?		0 (0%)	0 (0%)

## Discussion

Strategies to reduce surgical morbidity while providing durable benefits are critical for patients undergoing surgical correction for symptomatic CM1. Our case series describes how sparing durotomy in conjunction with intraoperative ultrasound verification of good CSF flow provides similar clinical and radiographic outcomes to durotomy in an adult population. Notably, these outcomes were coupled with reduced operative time and length of ICU stay. Given these findings, our results add to the emerging literature suggesting that in adult patients, durotomy-sparing decompression in patients with good CSF flow after bony decompression may on balance be the superior surgical procedure. 

The diagnosis of CM1 is common in clinical practice, hence justifying the efforts to find an optimal solution. The surgical treatment of CM1 aims to optimally facilitate CSF dynamics by restoring the subarachnoid spaces and the cisterna magna, decompress neural structures in the craniocervical junction, reduce any concurrent syrinxes, and ultimately improve or eliminate suboccipital headaches and neurologic deficits [17,21]. Historically for SOC, adding duraplasty has been predominantly the choice of procedure, with 92% of studies in the literature carrying out dural opening [3]. The majority of the evidence is in the pediatric population, where patients who received dural opening generally have better symptom improvement, syrinx reduction, and lower revision rates, at the cost of longer operating times and higher complication rates [22,23]. Given the success of duraplasty in children, duraplasty has also been used in the adult population in similar frequencies [3]. Nevertheless, there is a difference in the disease presentation, severity, and potential existing compensation mechanisms between pediatric and adult CM1 patients. The optimal surgical modality is yet to be clearly defined in the adult population, as original studies and meta-analyses have not yet clearly identified the superiority of duraplasty or decompression alone [16]. 

The absence of clear data on the pathophysiology and symptomatology of the disease, coupled with a lack of standardized methods on the surgical technique and post-operative outcome measurement, has clouded robust conclusions [16,24]. Further clouding these comparisons are the predominance of analyses on children only, or mixed cohorts with adults and children. In comparison to children who have more varied symptomology, adults classically present with strain headaches, which occur in 80-100% of patients [25]. Furthermore, adults often present at a later stage of their disease due to a possibly less-compromised CSF circulation. CSF leakage issues are likely different in the adult population, due to differences in muscle and subcutaneous tissue coverage [4]. Neurologic outcomes have also been known to be different in the two age cohorts, where adults had significantly worse neurologic outcomes, with respect to resolution of headaches and improvements in neurologic deficits [3]. These potentially important differences suggest that the optimal treatment for adult CM1 patients might be different from the optimal treatment for pediatric CM1 patients and justify separate analyses in the adult population.

Most of the existing studies comparing decompression alone with dural opening do not have a selection criterion for which patient should receive which treatment modality. However, the use of intraoperative ultrasound could help surgeons ascertain if bony decompression alone might be sufficient for each case. Using intraoperative ultrasound to confirm the re-expansion of the cisterna magna, optimal cerebellar tonsillar pulsations, and proper CSF flow across the craniometrical junction after decompression can assist in evaluating the need for dural opening. If these goals are robustly achieved, our evidence in conjunction with existing studies [15,26] suggests that duraplasty is not required. Hence, each operation can be tailored to each case, thereby ensuring surgical efficacy while limiting morbidity.

In our clinically and radiographically similar sets of patients, clinical outcomes, as measured via CCOS and CSS scores and radiographic evaluations of cerebellar ectopia and syrinx characteristics, were statistically similar between our two cohorts. Though rationales and outcome data exist in favor of both duraplasty and dura-sparing SOC, our findings suggest indifference at three years of follow-up. Relaxation of the posterior cerebellar fossa content is reportedly the best radiologic predictor of symptom improvement [27]. Herein lies the rationale for duraplasty, which aims to recreate the cisterna magna directly via an expansion duraplasty [16]. We reported resolved or improved symptoms in 85.7% of duraplasty cases. Consistent with our findings, improvement in neurologic symptoms seems to stabilize around 85% in the long-term period with duraplasty in the literature [16], which in some reports has an improved relative risk (RR) of 1.24 when compared to duraplasty-sparing procedure, although this is reported in a mixture of pediatric and adult patients [28]. This good rate of clinical improvement is coupled with a low reported rate of revision surgery, though balanced with longer hospital stays, costs, and complications [4].

The rationale for decompression without duraplasty is that the bony decompression of the suboccipital squama and atlas alone is potentially sufficient to allow the recreation of cisterna magna [29]. This technique is being increasingly used in conjunction with intraoperative ultrasound, as we did in our study, where high-frequency probes with color Doppler allow for clear visualization of the anatomy and flow measurements. In our case series, we found that 100% of the patients had their clinical symptoms improve or resolve following decompression without duraplasty, which was not significantly different from the duraplasty cohort. In the literature, some historical series report the success rate at 86-97% [16]. One recent meta-analysis of adult patients identified a success rate of 76%, which also was not statistically different than the duraplasty patients [17]. Similar to our findings, other studies have corroborated this lack of significant difference between the groups [4,11]. Thus, with respect to symptom improvement, though there are some conflicting studies, recent evidence is emerging toward no statistical difference between the duraplasty and duraplasty-sparing cohorts, especially in the adult population.

Hence, the rationale for duraplasty sparing stems not from enhanced symptom improvement but rather from suspected virtues in other areas, including requiring fewer hospital resources and a lesser complication rate [4,30]. We identified that on average, the duraplasty cohort required surgical times that were almost double the duraplasty sparing cohort. In addition, they on average spent an additional day in the ICU. In corroboration with our findings, Limondai et al. reported significantly decreased operative time, duration of hospitalization, and hospital charges in their duraplasty-sparing cohort [31]. With respect to complications, we found no differences between the groups, as no major medical or surgical complications were found in any patient. Complications rates in the literature generally center at 4-5% [3], with CSF-related events constituting the majority in duraplasty cases [12]. Complications associated with dural opening include CSF leakage, bleeding from dural sinuses, scarring in the subarachnoid space, meningitis, symptomatic pseudomeningoceles, and dural ectasia, all of which may require re-operation in some cases [32]. In the adult population specifically, a meta-analysis identified the most frequent complications as CSF fistula and infection [17]. Most studies have found a higher rate of complication in duraplasty cohorts [4,33,34]. The complication rate of duraplasty is around 15% [16], with risk ratios of 5.23 and 4.02 for CSF leaks and meningitis, respectively, when compared to duraplasty-sparing procedures [11]. Other meta-analyses have shown the overall RR of any complication to be 3.79, CSF leak to be 9.74, and neurologic deficit to be 8.76 [28]. Hence, dura opening and duraplasty after SOC are unequivocally burdened by increased resource utilization and complication rate.

The main causes of revision surgery following SOC decompression for CM1 are inadequate initial decompression and/or the occurrence of postoperative complications [35]. Though we found no cases of revision surgery in either cohort, the literature remains conflicted with respect to reoperation rates between the cohorts. One meta-analysis reports no significant differences in the reoperation rates [12], though other reports suggest that the risk of re-operation is lower with duraplasty, with an RR of around 0.12-0.15 [4,11], which correlates to a risk of around 2-3.5% for duraplasty and 6-7% for duraplasty sparing [16]. However, in the cases where complications do exist, especially CSF-related complications, the risk of re-operation is 25 times greater in the duraplasty cohort [36]. One notable consideration to account for these differences in re-operation rates is the premise of the unused treatment option. When only decompression is preformed, reoperation with duraplasty remains an option, which may inflate the number of re-operations due to clinical failure. If adequate decompression with duraplasty is initially performed, and surgical failure is encountered, further interventions are less likely to be offered [4]. Additionally, intraoperative ultrasound can help in assessing the correct extension of craniectomy and/or laminectomy. Hence, with proper intra-operative ultrasound confirmation of adequate decompression and restoration of CSF flow, the likelihood of re-operation due to initial inadequate decompression should be minimized.

This study has several limitations, many of which are inherent to small retrospective case series analyses. We examined patients from a single surgeon (YL), and therefore the number of patients in our cohorts was limited. We did not exclude patients with previous decompression due to low case numbers, but this could have potentially biased the selection. Hence, this restricted the statistical power of our analyses, which could have more robustly corroborated or disagreed with the findings in the literature. In particular, the number of patients in our study precluded analysis of the effect of duraplasty on spinal syrinx, though our data hint that syrinxes may resolve better with duraplasty. We noted a trend of duraplasty patients reporting higher pain and pain-medication usage, both immediately post-operatively and at one month follow-up, yet this was limited by the small sample size in reaching statistical significance. Our follow-up was limited to three years on average, which may not capture the full-scope of possible stable long-term clinical and radiographic outcomes. Finally, though we used retrospective analyses to identify clinical outcomes on CCOS and CSS scores, prospective, more thorough, standardized outcome scaling could allow for more robust conclusions. Similarly, given the retrospective nature of the data, we were unable to quantitatively characterize sufficient tonsilar pulsatility, and instead relied on the expertise of the senior surgeon. Prospective studies should aim to replicate these findings in a larger cohort, with a systematic method of assessing sufficient CSF flow.

## Conclusions

Dural opening and duraplasty are often carried out in both pediatric and adult cohorts for the treatment of CM1, which is thought to be necessary for adequate decompression at the cost of heightened surgical complications. However, the need for duraplasty in the adult cohort remains unclear. Our findings suggest that SOC without dura opening and duraplasty may be a viable surgical approach in the treatment of CM1 in adults, particularly when intraoperative ultrasound confirms adequate restoration of CSF flow. Our findings indicate that this approach yields similar clinical and radiographic outcomes in treating symptomatic CM1 compared to SOC with durotomy and duraplasty, while significantly reducing operative time and length of ICU stay, thus providing lower surgical morbidities. Further prospective studies with larger cohorts and longer follow-up periods are warranted to validate these findings and refine treatment guidelines for this patient population.
